# Identifying the Minimal Clinically Important Difference in Emotion Regulation Among Youth Using the JoyPop App: Survey Study

**DOI:** 10.2196/64483

**Published:** 2025-01-23

**Authors:** Jaidyn Charlton, Ishaq Malik, Angela M Ashley, Amanda Newton, Elaine Toombs, Fred Schmidt, Janine V Olthuis, Kristine Stasiuk, Tina Bobinski, Aislin Mushquash

**Affiliations:** 1 Department of Psychology Lakehead University Thunder Bay, ON Canada; 2 Department of Pediatrics University of Alberta Edmonton, AB Canada; 3 Department of Psychology University of New Brunswick Fredericton, NB Canada; 4 Dilico Anishinabek Family Care Fort William First Nation, ON Canada

**Keywords:** mHealth, mobile health, app, psychometrics, emotion regulation, Indigenous mental health, Indigenous youth, mental health interventions, resilience, clinical psychology, adolescent mental health, mental health, JoyPop, pediatrics, mobile phone

## Abstract

**Background:**

The minimal clinically important difference (MCID) is an important threshold to consider when evaluating the meaningfulness of improvement following an intervention. The JoyPop app is an evidence-based smartphone app designed to improve resilience and emotion regulation. Information is needed regarding the JoyPop app’s MCID among culturally diverse youth.

**Objective:**

This study aims to calculate the MCID for youth using the JoyPop app and to explore how the MCID may differ for a subset of Indigenous youth.

**Methods:**

Youth (N=36; aged 12-18 years) were recruited to use the JoyPop app for up to 4 weeks as part of a larger pilot evaluation. Results were based on measures completed after 2 weeks of app use. The MCID was calculated using emotion regulation change scores (Difficulties in Emotion Regulation–Short Form [DERS-SF]) and subjective ratings on the Global Rating of Change Scale (GRCS). This MCID calculation was completed for youth overall and separately for Indigenous youth only.

**Results:**

A significant correlation between GRCS scores and change scores on the DERS-SF supported face validity (*r*=–0.37; *P*=.04). The MCID in emotion regulation following the use of the JoyPop app for youth overall was 2.80 on the DERS-SF. The MCID for Indigenous youth was 4.29 on the DERS-SF. In addition, most youth reported improved emotion regulation after using the JoyPop app.

**Conclusions:**

These MCID findings provide a meaningful threshold for improvement in emotion regulation for the JoyPop app. They provide potential effect sizes and can aid in sample size estimations for future research with the JoyPop app or e-mental health technologies in general. The difference between overall youth and Indigenous youth MCID values also highlights the importance of patient-oriented ratings of symptom improvement as well as cultural considerations when conducting intervention research and monitoring new interventions in clinical practice.

## Introduction

The minimal clinically important difference (MCID) is the smallest change in an outcome (eg, symptom or ability) that patients consider meaningful to continue using an intervention [1]. These patient-derived scores serve as thresholds for outcome improvement when evaluating new interventions [1] and inform researchers on potential effect sizes in clinical trials.

**Figure 1:**
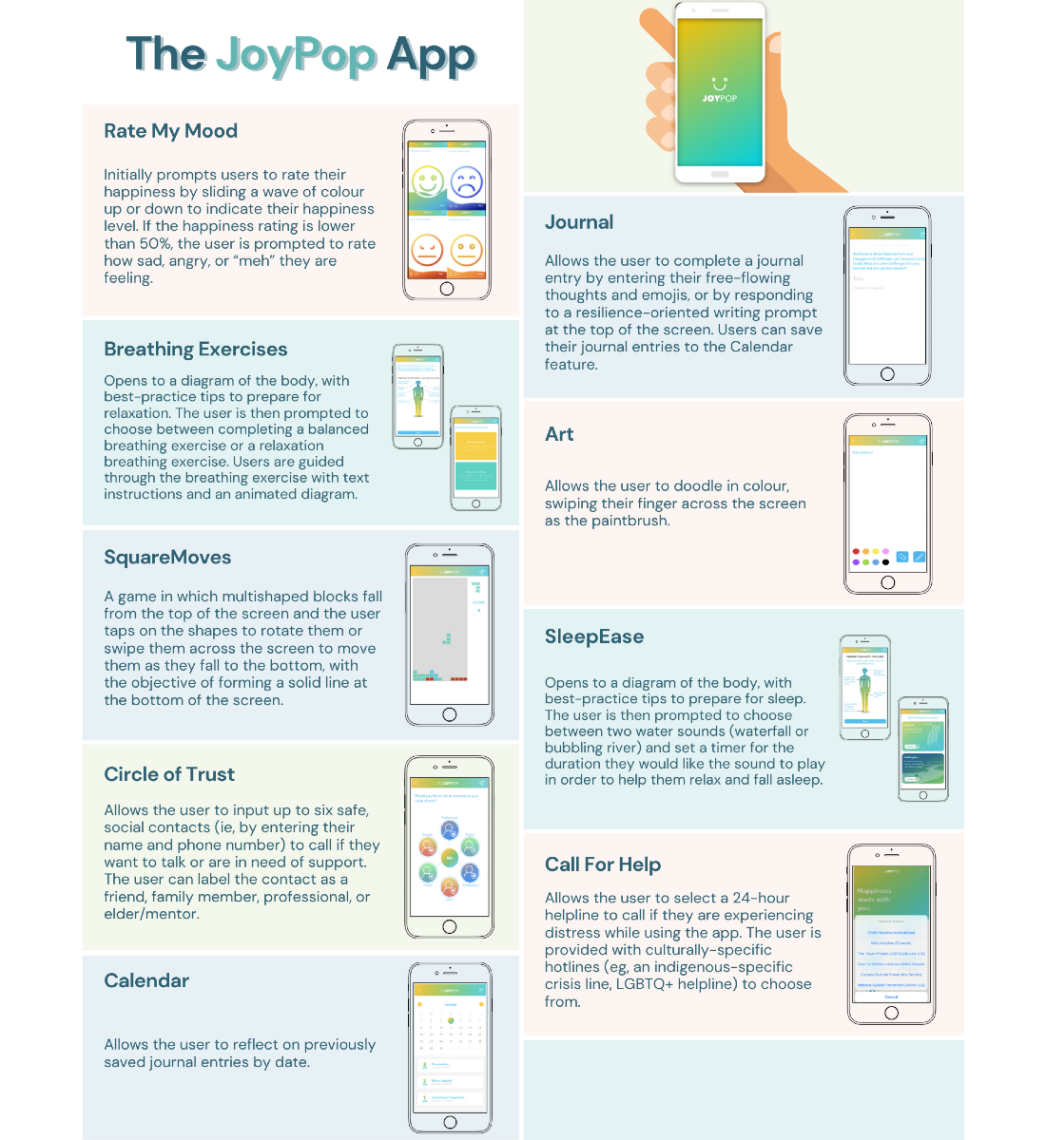
JoyPop app features.

The JoyPop app is a mobile health (mHealth) tool with a growing evidence base supporting its use among youth (Figure 1) [2]. JoyPop app use is associated with improved emotion regulation [2], and service providers, adolescents, and young adults view it as an acceptable, useful, and feasible tool to support existing mental health services [3,4]. Research to determine how much change in emotion regulation is clinically meaningful to app users is needed. Additionally, identifying the MCID among Indigenous youth, who experience greater mental health difficulties [5], is required.

We asked youth about the emotion regulation change that was important to them before and after using the JoyPop app. We calculated the MCID for all youth and Indigenous youth. We also explored the importance of patient perspectives and how MCIDs may differ between populations to provide patient-oriented recommendations for future research. Given this study’s exploratory nature, we did not have a priori hypotheses.

## Methods

### Participants and Procedure

All youth (aged 12-18 years) who used the JoyPop app while receiving mental health services from the 2 largest mental health organizations in Northwestern Ontario were eligible to participate; 36 participated. Staff (ie, the youth’s clinician) from the organizations informed them about the study. Staff sent the research team contact information for youth who were interested. Youth were contacted to receive further information. Interested youth attended an orientation session and downloaded the app onto their own device or were provided with a refurbished phone (containing just the JoyPop app) to use during the study. Participants were encouraged to use the app at least twice daily for 4 weeks and were sent 2 daily reminders to use the app through email and text. Participants completed measures for a larger pilot study at baseline (time 1; N=36), after 2 weeks (time 2; n=31), and after 4 weeks (time 3; n=25). Compensation was provided for each time point completed (up to CAD $80 [US $54]). If the JoyPop app was downloaded to participants’ devices, they could continue using it after study completion. Given the pilot study’s small sample size and attrition by time 3, we used time-1 and time-2 data for calculating the MCID.

### Measures

#### GRCS

The Global Rating of Change Scale (GRCS) is a single-item measure assessing the degree of change individuals felt occurred during or after an intervention [6]. The GRCS asked participants “As a result of using the JoyPop app, has your ability to understand and manage your emotions been...” Participants responded using an 11-point Likert scale (–5=“a very great deal worse” to 0=“no change” to +5=“a very great deal better”) at time 2. At time 1, we asked participants how much change in their ability to regulate their emotions they “would like to see” to deem using the app worthwhile.

#### DERS-SF

The Difficulties in Emotion Regulation Scale–Short Form (DERS-SF) [7,8] is an 18-item questionnaire assessing emotion regulation abilities (eg, “When I was upset, I became out of control”) rated on a 5-point Likert scale (1=“almost never” to 5=“almost always”). Total scores range from 18 to 80, with higher scores indicating greater difficulties. The DERS-SF has satisfactory reliability and validity [7,8]. The reliability among participants in this study was excellent (time-1 α=.90; time-2 α=.94).

### Statistical Analysis

We used the anchor-based method: patient-reported scores on outcome measures were compared with a more subjective measure of change (“anchor” or cutoff) [1]. Time-1 GRCS scores showed that most participants reported wanting to feel +2 “somewhat better” emotion regulation from using the app (Table 1). Therefore, +2 was used as the “anchor” at time 2 when determining the MCID. We calculated the mean change score on the DERS-SF (outcome measure) for participants who rated +2 on the GRCS (“anchor”)—this became the MCID. We ran descriptive statistics to calculate the proportion of participants who scored at or above the MCID. We completed these analyses for the total sample and separately for Indigenous participants. Correlation tested face validity.

**Table 1:** Frequencies of Global Rating of Change Scale (GRCS) ratings.

GRCS rating^a^	Time 1^b^ (N=36), n (%)	Time 2 (n=31), n (%)
Emotion regulation worsened (rated from –1 to –5)	4 (11)	2 (6)
No change in emotion regulation (rated 0)	4 (11)	5 (16)
Emotion regulation was almost the same, hardly better at all (rated +1)	3 (8)	8 (26)
Emotion regulation was somewhat better (rated +2)	13 (36)	10 (32)
Emotion regulation was much better (rated from +3 to +5)	12 (33)	6 (19)

^a^The percentage of youth that rated each item on the GRCS (N=36). Participants (aged 12-18 years) were recruited for the pilot study from 2 mental health organizations in Northwestern Ontario.

^b^GRCS ratings at time 1 (N=36) reflect how much change youth prospectively wanted to see from using the JoyPop app. GRCS ratings at time 2 (n=31) reflect how much change youth actually experienced after 2 weeks of using the JoyPop app.

### Ethical Considerations

The study was reviewed and approved by research ethics boards at Lakehead University (ROMEO #1468701) and the collaborating mental health organizations. Study information was reviewed with all youth. All participants provided informed consent. Data only contained the ID number provided at study onset. The list linking participant ID numbers to participant names was kept separately from the study dataset and was destroyed after data collection. Youth could withdraw at any time without penalty.

## Results

### Descriptive Statistics

Participant demographics (Table 2) and descriptive statistics for emotion regulation and global ratings of change (Table 3) are shown. Time-2 GRCS scores showed that many participants reported improved emotion regulation after using the app for 2 weeks (Table 1). A significant negative correlation between time-2 GRCS ratings and DERS-SF change scores provided face validity that the GRCS can indicate a meaningful patient-rated change in emotion regulation (*r*=–0.37; *P*=.04).

**Table 2:** Youth^a^ participant demographics.

Variables		Value (N=36)
**Ethnicity, n (%)**		
	White	9 (25)
	Indigenous	25 (69)
	Mixed	1 (3)
	South Asian	1 (3)
**Age (years), mean (SD)**		14.47 (1.75)
**Gender identity, n (%)**		
	Girl	22 (61)
	Boy	5 (14)
	Agender or nonbinary	7 (19)
	Transgender	2 (6)

^a^Participants (N=36; aged 12-18 years) were recruited for the pilot study from 2 mental health organizations in Northwestern Ontario.

**Table 3:** Mean and SD values of the Difficulties in Emotion Regulation Scale–Short Form (DERS-SF) and Global Rating of Change Scale (GRCS) overall and for Indigenous youth.

Participants^a^ and time point		Participants, n	DERS-SF^b^, mean (SD)	GRCS^c^, mean (SD)
**All youth**				
	Time 1	36	59.64 (14.03)	1.75 (1.54)
	Time 2	31	55.84 (16.11)	1.48 (1.44)
**Indigenous youth**				
	Time 1	25	58.44 (15.92)	1.64 (1.29)
	Time 2	21	53.19 (17.49)	1.43 (1.36)

^a^Participants (aged 12-18 years) were recruited for the pilot study from 2 mental health organizations in Northwestern Ontario.

^b^Higher scores indicate greater emotional dysregulation and lower scores indicate improvement.

^c^Higher scores indicate higher subjective improvement. GRCS ratings at time 1 reflect how much change youth prospectively wanted to see from using the JoyPop app. GRCS ratings at time 2 reflect how much change youth actually experienced after 2 weeks of using the JoyPop app.

### MCID

The mean change score on the DERS-SF for participants who rated +2 “somewhat better” on the GRCS at time 2 was –2.80 (SD 9.22), translating to an MCID of 2.80. Using this threshold, 45% (14/31) of participants met or exceeded this change after 2 weeks, regardless of their time-2 GRCS rating (Table 4). For Indigenous youth who rated +2 “somewhat better” on the GRCS, the mean change score on the DERS-SF was –4.29 (SD 8.96), indicating that they experienced a 4.29-point improvement in emotion regulation after using the app for 2 weeks and had an MCID of 4.29. Using this threshold, 43% (9/21) of Indigenous youth met or exceeded this change after 2 weeks.

**Table 4:** Frequencies of Global Rating of Change Scale (GRCS) ratings of those who met or exceeded the minimal clinically important difference on the Difficulties in Emotion Regulation Scale–Short Form.

GRCS rating^a^	Youth (n=14), n (%)
Emotion regulation worsened (rated from –1 to –5)	0
No change in emotion regulation (rated 0)	1 (7)
Emotion regulation was almost the same, hardly better at all (rated +1)	5 (36)
Emotion regulation was somewhat better (rated +2)	5 (36)
Emotion regulation was much better (rated from +3 to +5)	3 (21)

^a^Participants (aged 12-18 years) were recruited for the pilot study from 2 mental health organizations in Northwestern Ontario. This table represents only the youth who reached the minimal clinically important difference of 2.80 or exceeded it (14/31, 45%) and what they rated on the GCRS at time 2.

## Discussion

The MCID in emotion regulation was 2.80 for all youth and 4.29 for Indigenous youth. These provide preliminary effect sizes for future research as benchmarks for meaningful patient-oriented change, rather than solely basing findings on statistical significance, which lacks practicality or patient-oriented relevance [9]. Future study designs could recognize that youth generally need at least a 2.80-point improvement in their emotion regulation on the DERS-SF to consider the JoyPop app worthwhile. These findings can also inform future sample size calculations for JoyPop research [10,11].

Evaluating the MCID among Indigenous youth allowed us to consider their unique cultural experiences and provided insight into the effectiveness and appropriateness of the app for this subgroup. Our results suggest that Indigenous youth need to see a greater improvement in emotion regulation to deem the JoyPop app worthwhile and to start feeling “somewhat better.” Conducting MCID research with different groups, beyond ethnicity, can provide information on distinct change thresholds that interventions should aim for as opposed to “one-size-fits-all” approaches.

The findings also highlight the value of pairing outcome measures with patient-rated measures of change [12]. Not all participants who met or exceeded the MCID threshold subjectively rated an improvement in their emotion regulation (Table 4). This suggests that many, but not all, youth notice improvements in their emotion regulation, and that some youth require a higher threshold of change (>2.80) to notice improvement. This underscores the importance of measuring how individuals perceive their symptoms and abilities in relation to an intervention. If a young person does not perceive improvement from using an intervention, they are unlikely to continue, even if outcome data show improvement. We recommend subjective experience ratings (eg, GRCS) alongside outcome measures (eg, DERS-SF) when evaluating mHealth interventions.

MCID research typically involves participants reporting on what happened following an intervention. Previous research has relied on clinician judgment and clinical theory to determine their “anchor” on which to base the MCID [13]. We included a novel prospective question at time 1 to explore what youth would want to experience to inform the choice of anchor for our sample. Most youth wanted to see a minimum “somewhat better” improvement in their emotion regulation, aligning with previous research using +2 as an anchor [13]. This is useful in promoting the JoyPop app to youth and service providers as it highlights continuity between clinician and client judgment.

Despite encouraging preliminary findings, this study has limitations. The GRCS has received criticism as a single-item measure, which lacks robust psychometric properties [14]. Single-item measures do not capture the complexity of experience and can introduce differences in interpretation between participants [15]. However, single-item measures are informative and valid when used appropriately, such as concurrently with multi-item measures (eg, alongside the DERS-SF) or when they ask straightforward questions that do not require multiple items (eg, how much change in emotion regulation youth experienced) [14]. Another limitation is that the MCIDs were calculated based on a small sample, which limits generalizability. Nevertheless, these findings provide valuable contributions to the JoyPop app’s evidence base. Through cumulative longitudinal evidence, the MCIDs can be improved and expanded on for different populations (eg, adolescent vs young adult; differing ethnicities), thus demonstrating a client-centered approach to research and mHealth app implementation [16].

## Abbreviations

DERS-SF: Difficulties in Emotion Regulation Scale–Short Form
GRCS: Global Rating of Change Scale
MCID: minimal clinically important difference
mHealth: mobile health

## Data Availability

The dataset generated and analyzed is publicly available due to our team’s adherence to the Ownership, Control, Access, and Possession (OCAP) standards set by the First Nations Information Governance Centre. Questions about study datasets can be directed to the corresponding author and principal investigator (ARM).
